# Supplementary material for: Designing Born-Accessible Courses in Data Science and Visualization: Challenges and Opportunities of a Remote Curriculum Taught by Blind Instructors to Blind Students
Source: Eurograph IEEE VGTC Symp Vis. Author manuscript; Available in PMC 2025 Mar 2. (PMC11872190)
Supplement: Appendix [file NIHMS2025217-supplement-Appendix.pdf]

Designing Born-Accessible Courses in Data Science and Visualization: Challenges and Opportunities of a Remote Curriculum Taught by Blind Instructors to Blind Students

JooYoung Seo[1], Sile O'Modhrain[2], Yilin Xia[1], Sanchita Kamath[1], Bongshin Lee[3], and James Coughlan[4]

[1]University of Illinois Urbana-Champaign, School of Information Sciences, USA
[2]University of Michigan, School of Information, USA
[3]Yonsei University, Republic of Korea
[4]Smith-Kettlewell Eye Research Institute, USA

Abstract

While recent years have seen a growing interest in accessible visualization tools and techniques for blind people, little attention is paid to the learning opportunities and teaching strategies of data science and visualization tailored for blind individuals. Whereas the former focuses on the accessibility issues of data visualization tools, the latter is concerned with the learnability of concepts and skills for data science and visualization. In this paper, we present novel approaches to teaching data science and visualization to blind students in an online setting. Taught by blind instructors, nine blind learners having a wide range of professional backgrounds participated in a two-week summer course. We describe the course design, teaching strategies, and learning outcomes. We also discuss the challenges and opportunities of teaching data science and visualization to blind students. Our work contributes to the growing body of knowledge on accessible data science and visualization education, and provides insights into the design of online courses for blind students.

CCS Concepts
• ***Applied computing*** $\rightarrow$ ***Education;***

1. Research Questions

Our research questions aim to understand the effects of teaching data visualization techniques to blind individuals. We will use the pre- and post-surveys to address these key questions:

1. How does teaching data visualization techniques to blind individuals impact their confidence in data visualization?

2. Does a course on data visualization techniques improve the sense of agency in learning for blind individuals?

3. What is the experience of blind individuals with data visualization before the course?

4. How does prior experience with specific visualizations (such as barplot, heatmap, boxplot, and scatterplot) affect blind individuals' learning and confidence in using those visualizations?

5. How do blind individuals envision using data visualization techniques for their career development and professional growth?

6. Did the course on data visualization techniques meet the expectations of blind individuals in terms of their career development and professional growth?

7. What were the most challenging and rewarding aspects of the course for blind individuals in terms of their learning and professional growth?

8. How has the course on data visualization techniques impacted the career development and professional growth of blind individuals?

These research questions will guide our analysis and interpretation of the survey data to gain insights into the effects of teaching data visualization techniques to blind individuals.

2. Pre-Survey

2.1. Introduction

This survey aims to gauge your initial self-efficacy, sense of agency, and prior experiences with data science, data visualization, and specific visualizations. We also aim to understand more about your visual impairment, demographic information, screen reader products, refreshable braille display model, education level, learning goals, and career aspirations. Your responses will help us tailor the course to meet your needs. All information collected is confidential and used for educational and research purposes only.

© 2024 The Authors.
Proceedings published by Eurographics - The European Association for Computer Graphics.
This is an open access article under the terms of the Creative Commons Attribution License, which permits use, distribution and reproduction in any medium, provided the original work is properly cited.

2.1.1. Demographics

1. What is your age?: _______

2. Gender:
- [ ] Male
- [ ] Female
- [ ] Prefer not to say
- [ ] Other: _______

3. Occupation: _______
- Field: _______

4. Education Level:
- [ ] High School or equivalent
- [ ] Associate's Degree
- [ ] Bachelor's Degree
- [ ] Master's Degree
- [ ] Doctorate Degree
- [ ] Other: _______

5. Major: _______

6. Minor (if applicable):_______

2.1.2. Visual Impairment

7. How long have you been blind? _______

8. Can you describe your level of visual impairment in your left eye? _______

9. Can you describe your level of visual impairment in your right eye? _______

2.1.3. Assistive Technologies

10. What screen reader product(s) do you use?_______

11. How long have you been using this screen reader? _______

12. What model of refreshable braille display do you use? _______

13. How long have you been using this refreshable braille display?_______

2.1.4. Prior Experience

14. Have you had any experience with data science?
- [ ] Yes
- [ ] No

15. If yes, please briefly describe your experience with data science. _______

16. Have you had any experience with data visualization?
- [ ] Yes
- [ ] No

17. If yes, please briefly describe your experience with data visualization. _______

18. How have you been accessing data visualization? _______

19. Please score your level of confidence in creating the following types of visualizations on a scale of 1 to 5, with 1 being the lowest confidence and 5 being the highest confidence:
- Barplot: _______
- Heatmap: _______
- Boxplot: _______
- Scatterplot: _______

20. Please rate your level of confidence for interpreting each visualization on a scale of 1 to 5, with 1 being the lowest confidence and 5 being the highest confidence:
- Barplot: _______
- Heatmap: _______
- Boxplot: _______
- Scatterplot: _______

21. Have you used tactile representations (e.g., embossed or 3D graphs) of the following types of visualizations before?
- [ ] Barplot
- [ ] Heatmap
- [ ] Boxplot
- [ ] Scatterplot

22. For each of the following types of visualizations, how well do you believe you know what they look like? Please rate your level of knowledge (1- No knowledge at all, 5 - Completely knowledgeable):
- Barplot: _______
- Heatmap: _______
- Boxplot: _______
- Scatterplot: _______

23. Please rate your current level of computer programming skills (1- None, 5 - Very proficient): _______

24. How many years of programming experience do you have? _______

25. Have you used any of the following data science environments before? For each environment you've used, please specify how long you've been using it.
- [ ] R (Length of usage: _______)
- [ ] Python (Length of usage: _______)
- [ ] SPSS (Length of usage: _______)
- [ ] SAS (Length of usage: _______)
- [ ] MatLab (Length of usage: _______)
- [ ] Stata (Length of usage: _______)
- [ ] MiniTab (Length of usage: _______)
- [ ] Visual Studio Code (Length of usage: _______)
- [ ] Other (Specify: _______, Length of usage: _______)

26. What do you want to learn in this course? Please de-

© 2024 The Authors.

scribe your learning goals. _______

27. How do you envision using data visualization techniques for your career development or professional growth? _______

2.1.5. Preferred Modality for Learning

28. Which modality or combination of modalities do you prefer for learning data visualization techniques? Please select all that apply:
- [ ] Braille
- [ ] Sonification
- [ ] Text-to-speech

3. Post-Survey

3.1. Introduction

This survey aims to understand your self-efficacy, sense of agency, and experiences with data visualization after the course. We appreciate your honest feedback. Your responses will help us improve future sessions.

3.1.1. Self-efficacy and Sense of Agency

1. How much control do you feel you have over your learning process now? (1- No control at all, 5 - Complete control)
1 [ ] 2 [ ] 3 [ ] 4 [ ] 5 [ ]

3.1.2. Course Feedback

2. What was the most challenging aspect of the course for you? _______

3. What was the most rewarding part of the course for you? _______

4. On a scale of 1 to 5, how well did the course meet your expectations in terms of your career development or professional growth? _______

5. Please briefly explain your rating for the last question _______

6. How has this course impacted your career development or professional growth? _______

3.1.3. Visualization Confidence

7. How confident are you in your ability to create the following data visualization after this course? (1- Not at all confident, 5 - Very confident)
- Barplot: _______
- Heatmap: _______
- Boxplot: _______
- Scatterplot: _______

8. Please briefly explain your rating. _______

9. Please rate your level of confidence in interpreting each of the following visualizations now, after completing the course (1- Not at all confident, 5 - Very confident): - Barplot: _______
- Heatmap: _______
- Boxplot: _______
- Scatterplot: _______

© 2024 The Authors.
Proceedings published by Eurographics - The European Association for Computer Graphics.
